# Vegetable and Fruit Consumption Among Adolescents in the Gulf Cooperation Council Countries: A Pooled Analysis of National School Surveys

**DOI:** 10.3390/nu18172783

**Published:** 2026-08-26

**Authors:** Abdulmohsen Hamdan Al-Zalabani

**Affiliations:** Department of Family and Community Medicine, College of Medicine, Taibah University, Madinah 42353, Saudi Arabia; azalabani@taibahu.edu.sa

**Keywords:** adolescent, fruit, vegetables, diet, prevalence, health behavior, students, Middle East, cross-sectional studies

## Abstract

**Background/Objectives:** Insufficient vegetable and fruit (VF) consumption during adolescence increases the risk of diet-related non-communicable diseases, yet no pooled, survey-weighted analysis of VF intake exists for the Gulf Cooperation Council (GCC) countries. This study estimated the prevalence and correlates of VF consumption among adolescents in the GCC countries. **Methods:** Cross-sectional data from the most recent Global School-based Student Health Survey (GSHS) in five GCC countries—Bahrain (2016), Kuwait (2015), Oman (2015), Qatar (2011), and the United Arab Emirates (UAE; 2016)—were pooled (*n* = 21,528 school-going adolescents aged 12–18 years); GSHS data were not available for Saudi Arabia. The primary outcome was consuming VF five or more times per day. Because the GSHS items record the frequency of eating occasions and not portion size, this is a frequency-based proxy for the World Health Organization (WHO) recommendation of at least 400 g per day. Survey-weighted prevalence estimates with 95% confidence intervals (CIs) and adjusted prevalence ratios (aPRs) from Poisson regression models were computed, overall and by sex. **Results:** The weighted prevalence of consuming VF on five or more occasions per day ranged from 15.5% (95% CI: 13.2–18.1) in Kuwait to 25.2% (95% CI: 22.6–28.0) in Qatar and was higher among boys than girls in all five countries. The outcome was less prevalent at older ages (aPR = 0.66, 95% CI: 0.56–0.79 for 16–18 years vs. ≤12 years), among adolescents reporting low parental support (aPR = 0.85, 95% CI: 0.76–0.95), and among those who were not physically active daily (aPR = 0.68, 95% CI: 0.62–0.74) and was more prevalent among boys (aPR = 1.18, 95% CI: 1.08–1.29). **Conclusions:** Fewer than one in four adolescents in the GCC countries consumed VF on five or more occasions per day. Multicomponent school-based and family-engaging interventions are needed, particularly for girls and older adolescents.

## 1. Introduction

Adequate consumption of vegetables and fruit (VF) is a cornerstone of a healthy diet and a key protective factor against non-communicable diseases (NCDs). The World Health Organization (WHO) recommends a population-level intake of at least 400 g of VF per day, commonly operationalized as five or more servings per day [1]. The 2023 WHO guideline on carbohydrate intake retains the target of at least 400 g of VF per day and extends it, as a conditional recommendation, to children and adolescents from the age of 10 years [2]. Meta-analytic evidence indicates that higher VF consumption is associated with an approximately 24% lower risk of metabolic syndrome [3]. Adolescence is a critical developmental window during which dietary preferences and habits are established and tend to track into adulthood; nutritional choices made in this formative period can therefore shape an individual’s long-term health trajectory. Despite this, insufficient VF intake remains one of the most common unhealthy dietary behaviors among adolescents worldwide, frequently clustering with other risk behaviors such as physical inactivity and consumption of sugar-sweetened beverages and fast food [4].

The Gulf Cooperation Council (GCC) countries (Bahrain, Kuwait, Oman, Qatar, the United Arab Emirates (UAE), and Saudi Arabia) are undergoing a pronounced nutrition transition characterized by rapid urbanization, increased consumption of processed and energy-dense foods, and a shift away from traditional dietary patterns, accompanied by a rising burden of diet-related NCDs. Minimal consumption of VF has been explicitly identified as a documented adolescent health problem in the Gulf region [5]. A systematic review of 44 studies from Arab countries found that only 10–29% of adolescents met the recommendation of five or more VF servings per day, with fruit consumed less frequently than vegetables [6]. In Saudi Arabia, low VF consumption and frequent fast-food intake have been reported as dominant unhealthy dietary patterns among adolescents [7]. A mixed-methods study in Dubai found that only 28% of adolescents met VF recommendations, with family meals and parental support emerging as key predictors [8]. Analyses of Global School-based Student Health Survey (GSHS) data from the UAE have likewise documented persistently high levels of inadequate VF intake between 2005 and 2016 [9], and a recent meta-analysis from the Middle East and North Africa (MENA) region reported significant sex differences in dietary behaviors, with boys consuming vegetables more frequently than girls in most countries [10].

Nevertheless, important gaps remain. First, no pooled, survey-weighted GSHS analysis has focused specifically on VF outcomes in the GCC countries. Second, recent VF-focused analyses of the GSHS waves from Kuwait, Qatar, Bahrain, and Oman are lacking, as published analyses of these datasets have addressed other outcomes. Accordingly, this study aimed to estimate the survey-weighted prevalence of VF consumption and of consuming VF on five or more occasions per day, among school-going adolescents in the GCC countries, and to identify sociodemographic and behavioral correlates of this frequency-based indicator, overall and by sex, using nationally representative GSHS data. In doing so, it establishes a regional baseline that is comparable across countries and against which subsequent surveillance waves and national nutrition policies can be evaluated. It also identifies the subgroups toward which limited school- and family-based intervention resources can most usefully be directed.

## 2. Materials and Methods

This study is a pooled analysis of secondary data from the GSHS [11]. Due to overlap, portions of Section 2.1, Section 2.2, Section 2.3, Section 2.4 and Section 2.5 have been recycled, with minor modifications, from our previous publication using the same survey instrument and datasets [12,13], consistent with Text Recycling Research Project (TRRP) transparency guidance [14].

### 2.1. Study Design and Data Source

This pooled analysis study utilized data from the GSHS, a collaborative surveillance project between the WHO and the U.S. Centers for Disease Control and Prevention (CDC). The GSHS is a nationally representative, cross-sectional survey that collects data on health behaviors and health-related risk factors among adolescents in more than 100 countries worldwide [12].

### 2.2. Participants and Sampling

The current study included the most recent publicly available GSHS datasets from five GCC countries: Bahrain (2016), Kuwait (2015), Oman (2015), Qatar (2011), and the UAE (2016). Saudi Arabia was excluded because GSHS data were not available. In each country, a two-stage cluster sampling design was used to select a representative sample of students aged 12–18 years from public and private schools. The first stage involved selecting schools with a probability proportional to size, while the second stage involved randomly selecting classes within each school. All students in the selected classes were eligible to participate [12]. Records with missing data on the combined VF outcome were excluded, yielding a final analytic sample of 21,528 adolescents.

### 2.3. Measures

Outcome. VF intake was evaluated using one question for each, administered in identical wording in all five surveys: “During the past 30 days, how many times per day did you usually eat fruit, such as dates, bananas, apples, oranges, mangos, berries, or any other fruits?” and “During the past 30 days, how many times per day did you usually eat vegetables, such as lettuce, cucumbers, tomatoes, carrots, or any other vegetables?” Each item had seven ordered response options, ranging from “I did not eat fruit [vegetables] during the past 30 days” to “five or more times per day.” Each response was assigned its numeric daily frequency: “I did not eat fruit [vegetables] during the past 30 days” = 0; “less than 1 time per day” = 0.5; “1 time per day” = 1; “2 times per day” = 2; “3 times per day” = 3; “4 times per day” = 4; and “5 or more times per day” = 5.5. Because the highest category is open-ended and “less than 1 time per day” is not a point value, these two options were assigned 5.5 and 0.5, respectively. The VF values were summed to give a combined daily frequency, and this sum was dichotomized at ≥5 to indicate consumption of VF on five or more occasions per day (yes/no). For descriptive presentation only, VF consumption was collapsed into four categories: none; less than once to once per day; 2–3 times per day; and 4 or more times per day. These collapsed categories were not used to construct the outcome. The verbatim items and their full response options are set out in Supplementary Table S1. The binary indicator of consuming VF on five or more occasions per day served as the outcome in all multivariable models.

Covariates. Food insecurity, used as a proxy for low socioeconomic status, was measured using the question “During the past 30 days, how often did you go hungry because there was not enough food in your home?” and was categorized as never, rarely/sometimes, or most of the time/always. The GSHS contains no measure of household income, parental occupation, or parental education, so this single item is the only available proxy for socioeconomic position. Physical activity was assessed using the question “During the past 7 days, on how many days were you physically active for a total of at least 60 min per day?” and was dichotomized in the present study as being physically active for at least 60 min per day on all 7 days (yes/no). This dichotomy corresponds directly to the WHO recommendation of at least 60 min of physical activity daily and is the conventional GSHS indicator, which supports comparability with previous GSHS analyses. Because it groups adolescents who were active on some days with those who were active on none, an ordinal version was also examined in a sensitivity analysis (Section 2.4). Additional covariates included age group (≤12, 13–15, and 16–18 years), sex, soft drink consumption (0, <1, 1–2, or ≥3 times per day), fast food consumption (0, 1–3, or 4–7 days in the past week), and low parental support. Low parental support was determined based on three GSHS items that asked participants how often, during the past 30 days, their parents or guardians (a) checked whether their homework was completed, (b) understood their problems and worries, and (c) were aware of what they were doing during their free time. Adolescents who responded “rarely” or “never” to all three questions were classified as having low parental support; all others were classified as having adequate support [12].

### 2.4. Statistical Analysis

All analyses were conducted using Stata version 18.0 (StataCorp LLC, College Station, TX, USA). The complex survey design was accounted for using the svy commands, with the sampling weights, clustering (primary sampling units), and stratification variables provided by the GSHS [12]. Strata containing a single sampling unit were centered at the grand mean. Descriptive statistics summarized the unweighted characteristics of the study population, and survey-weighted percentages with 95% confidence intervals (CIs) were estimated for fruit consumption, vegetable consumption, and consumption of VF on five or more occasions per day, by country and by sex; sex-specific estimates used survey subpopulation estimation rather than case deletion. Associations between the outcome and the covariates were examined using Poisson regression models with a log link fitted within the survey framework, yielding adjusted prevalence ratios (aPRs) with 95% CIs. Models were adjusted for country, age group, sex, food insecurity, physical activity, fast food consumption, soft drink consumption, and low parental support, and separate models were fitted for the overall sample and stratified by sex. An interaction term between sex and low parental support was fitted in the pooled model and tested formally. To assess whether differences between countries reflected their differing age structures, prevalence was additionally estimated by direct standardization to the pooled five-country weighted age distribution. In a sensitivity analysis, the dichotomous physical activity variable was replaced by an ordinal version (0, 1–2, 3–4, 5–6, and 7 active days) and by a continuous term for the number of active days (Supplementary Table S3). Multicollinearity among covariates was assessed.

### 2.5. Ethics

This study analyzed existing, de-identified public-use data from the GSHS. In each participating country, the survey was approved by the relevant Institutional Review Board, and student participation was voluntary and anonymous, with consent procedures implemented according to each country’s protocol.

## 3. Results

### 3.1. Sample Characteristics

The final sample included 21,528 adolescents from five GCC countries: Bahrain (*n* = 7037; 32.7%), Kuwait (*n* = 3534; 16.4%), Oman (*n* = 3376; 15.7%), Qatar (*n* = 1860; 8.6%), and the UAE (*n* = 5721; 26.6%). Overall, 57.1% of participants were aged 13–15 years and 51.7% were girls (Table 1). Nearly half of the participants (47.4%) reported going hungry at least rarely or sometimes in the past 30 days, including 8.3% who reported going hungry most of the time or always. Low parental support was reported by 13.6% of adolescents, ranging from 8.6% in Bahrain to 31.4% in Qatar. Only 16.1% were physically active for at least 60 min per day on all 7 days. Frequent fast-food consumption (4–7 days per week) was reported by 14.6%, and soft drink consumption three or more times per day was reported by 12.8%.

### 3.2. Prevalence of Vegetables and Fruit Consumption

Table 2 presents the survey-weighted prevalence of VF consumption frequency and of consuming VF on five or more occasions per day, by country and sex. The weighted prevalence of consuming VF on five or more occasions per day ranged from 15.5% (95% CI: 13.2–18.1) in Kuwait to 25.2% (95% CI: 22.6–28.0) in Qatar, with intermediate estimates in Bahrain (21.0%, 95% CI: 19.2–22.9), Oman (21.5%, 95% CI: 19.2–23.9), and the UAE (22.1%, 95% CI: 19.5–25.0). In all five countries, boys were more likely than girls to reach this threshold: prevalence among boys ranged from 15.9% (95% CI: 13.0–19.2) in Kuwait to 28.2% (95% CI: 24.4–32.3) in Qatar, compared with 14.8% (95% CI: 11.9–18.2) to 22.6% (95% CI: 19.3–26.2) among girls in the same countries. Age standardization to the pooled five-country weighted age distribution changed these estimates only slightly (Bahrain 19.9%, Kuwait 15.7%, Oman 21.7%, Qatar 26.6%, and the UAE 22.1%) (Supplementary Table S2).

Regarding fruit consumption, the proportion of adolescents who reported not eating any fruit in the past 30 days was highest in Qatar (16.2%, 95% CI: 14.2–18.4) and Kuwait (14.1%, 95% CI: 11.9–16.7) and lowest in Oman (5.4%, 95% CI: 4.2–6.9), the UAE (5.9%, 95% CI: 4.9–7.0), and Bahrain (6.0%, 95% CI: 5.2–7.0). In every country, the most common pattern was consuming fruit once per day or less, ranging from 45.6% (95% CI: 42.8–48.4) in Qatar to 61.7% (95% CI: 59.7–63.7) in Bahrain, whereas fruit consumption four or more times per day ranged from 4.8% (95% CI: 4.1–5.8) in Kuwait to 14.2% (95% CI: 12.3–16.4) in Qatar.

Vegetable consumption followed a broadly similar pattern. The proportion of adolescents who reported not eating any vegetables in the past 30 days was highest in Qatar (15.4%, 95% CI: 13.4–17.6) and lowest in the UAE (6.5%, 95% CI: 5.7–7.4), and consuming vegetables once per day or less was again the most common pattern in every country, ranging from 47.5% (95% CI: 44.5–50.4) in Qatar to 59.7% (95% CI: 57.0–62.3) in Oman. Vegetable consumption four or more times per day ranged from 7.6% (95% CI: 6.5–8.7) in Kuwait to 13.0% (95% CI: 10.8–15.5) in Qatar. In Kuwait, the proportion reporting no fruit consumption (14.1%) was notably higher than the proportion reporting no vegetable consumption (9.4%), whereas the two proportions were more similar in the other countries. High-frequency fruit consumption (≥4 times per day) was more prevalent among boys than girls in all five countries (e.g., 12.4% vs. 8.9% in Bahrain and 14.4% vs. 9.3% in Oman).

### 3.3. Correlates of Consuming Vegetables and Fruit on Five or More Occasions per Day

Table 3 presents the adjusted prevalence ratios for consuming VF on five or more occasions per day in the overall sample and stratified by sex; all models were additionally adjusted for country. The outcome was less prevalent at older ages. Compared with adolescents aged 12 years or younger, the adjusted prevalence was 17% lower among those aged 13–15 years (aPR = 0.83, 95% CI: 0.72–0.97) and 34% lower among those aged 16–18 years (aPR = 0.66, 95% CI: 0.56–0.79), with a similar gradient in both sexes. Boys had an 18% higher adjusted prevalence than girls (aPR = 1.18, 95% CI: 1.08–1.29). Food insecurity was not significantly associated with the outcome in the overall sample or in either sex.

Compared with adolescents who were physically active for at least 60 min per day on all 7 days, those who were not had a lower adjusted prevalence of the outcome (aPR = 0.68, 95% CI: 0.62–0.74), a pattern observed in both girls (aPR = 0.64) and boys (aPR = 0.71). In the sensitivity analysis using the ordinal measure of physical activity, the association was graded rather than a threshold effect: relative to activity on all 7 days, the adjusted prevalence ratio was 0.97 (95% CI: 0.86–1.10) for 5–6 days, 0.87 (0.78–0.97) for 3–4 days, 0.58 (0.52–0.65) for 1–2 days, and 0.53 (0.47–0.61) for no active days, corresponding to 1.10 (1.09–1.12) per additional active day; all other estimates were materially unchanged (Supplementary Table S3). Compared with adolescents who consumed no soft drinks, those consuming soft drinks less than once per day (aPR = 0.64, 95% CI: 0.59–0.70) and 1–2 times per day (aPR = 0.70, 95% CI: 0.64–0.78) had a lower prevalence of the outcome, whereas consumption three or more times per day was associated with a borderline higher prevalence (aPR = 1.13, 95% CI: 1.00–1.27). Fast food consumption was not associated with the outcome overall, although girls who consumed fast food on 4–7 days had a lower prevalence of the outcome (aPR = 0.82, 95% CI: 0.69–0.97). Low parental support was associated with a lower prevalence of the outcome overall (aPR = 0.85, 95% CI: 0.76–0.95); this association was pronounced among girls (aPR = 0.73, 95% CI: 0.61–0.86) but not evident among boys (aPR = 0.94, 95% CI: 0.81–1.08). The sex by low parental support interaction term was statistically significant (*p* = 0.019).

## 4. Discussion

This study provides the first pooled, survey-weighted estimates of VF consumption among adolescents across five GCC countries using nationally representative GSHS data. Fewer than one in four adolescents consumed VF five or more times per day, with the weighted prevalence ranging from 15.5% in Kuwait to 25.2% in Qatar. Boys were consistently more likely than girls to do so in every country, and the prevalence of the outcome declined markedly with age. Low parental support and lack of daily physical activity were independently associated with a lower prevalence of adequate VF consumption, whereas food insecurity showed no significant association.

The prevalence estimates observed here fall within the 10–29% range reported by a systematic review of VF consumption among adolescents in Arab countries [6], and are consistent with the 28% of Dubai adolescents meeting VF recommendations reported in an earlier mixed-methods study [8]. The tendency for fruit to be consumed less frequently than vegetables, most evident in Kuwait, where the proportion reporting no fruit consumption exceeded the proportion reporting no vegetable consumption, likewise echoes the Arab-region review [6]. They also align with trend analyses of UAE GSHS data documenting persistently high inadequate VF intake across the 2005, 2010, and 2016 survey waves [9], and with GSHS-based analyses from neighboring non-GCC settings such as Lebanon, where unhealthy dietary patterns were similarly widespread among school-going adolescents [15]. Comparable GSHS analyses outside the region, such as in Laos, have likewise found that only a minority of adolescents achieve adequate VF intake and that dietary risk behaviors cluster together [16]. Taken together, these findings indicate that inadequate VF consumption among GCC adolescents is not an isolated national phenomenon but a regional pattern consistent with the broader nutrition transition affecting Gulf societies [5,7], in which traditional diets are being displaced by energy-dense processed foods and beverages [17].

The WHO target of at least 400 g of VF per day is a quantity-based, population-level goal, and the simplified “five portions” message corresponds to approximately five 80 g portions. The GSHS items record the frequency of eating occasions and do not record portion size, so the indicator used here is a frequency-based proxy for that target rather than a measure of it. Agreement between the two depends on the average size of an eating occasion. In addition, WHO specifies that potatoes, cassava, and other starchy tubers should not be counted toward the target; the exemplar foods named in the GSHS items are all non-starchy, but the items close with “or any other fruits [vegetables]” and provide no food list, so this exclusion is not made explicit to respondents.

Differences between countries were modest and were not explained by the differing age structures of the samples. After direct standardization to the pooled five-country weighted age distribution, the estimates changed little. The lower prevalence in Kuwait was accompanied by the lowest prevalence of high-frequency fruit consumption and the highest proportion reporting no fruit consumption of any country, which suggests that the Kuwaiti difference is driven mainly by the fruit component. The surveys were also fielded in different years, and the Qatar survey predates the others by four to five years. Therefore, any secular change in consumption over that interval would be absorbed into the apparent differences between countries, and the Qatari estimate would be inflated relative to the others if consumption declined over that period.

The consistent male advantage observed in this study is in line with the MENA-wide GSHS meta-analysis, which found that boys consumed vegetables more frequently than girls in most countries [10]. Sex differences in health consciousness, body image concerns, and family food practices during adolescence may also contribute, and qualitative evidence from the region suggests that girls’ dietary behaviors are more closely tied to family meal routines and parental involvement [8]. The strong age gradient, with 16–18-year-olds one-third less likely to reach the threshold than the youngest adolescents, mirrors the widely documented deterioration of dietary quality across adolescence as autonomy over food choices increases and eating occasions shift away from the family environment.

The finding that low parental support was associated with a lower prevalence of the outcome among girls, but not among boys, adds GCC-specific evidence to a growing global literature. A multilevel analysis of GSHS data from 73 countries found that family support, peer support, and food security protect against the clustering of unhealthy lifestyle behaviors, including low VF intake [4], and parental support was a key predictor of VF intake among Emirati adolescents [8]. One interpretation is that adolescent girls in the region eat a larger share of their meals within the household, so that parental engagement translates more directly into what is available and eaten. In contrast, boys have greater discretionary access to food outside the home, where parental monitoring is less consequential for diet. A parallel sex difference was observed for frequent fast-food consumption, which was associated with lower consumption among girls only: where the household is the main setting for eating, its displacement by food from outside the home is more visible in the intake of girls. These interpretations are speculative because the GSHS does not measure meal location, food purchasing, or the content of parental oversight. In contrast, food insecurity was not significantly associated with VF consumption in this sample, which may reflect the relatively high-income context of the GCC countries, where the GSHS hunger item may capture transient or behavioral rather than economic food deprivation. Notably, in our previous analysis of the same datasets, the same food insecurity measure was strongly associated with frequent soft drink consumption [12], suggesting that socioeconomic disadvantage in this setting may shape beverage choices more than VF intake.

The lower prevalence of adequate VF consumption among adolescents who were not physically active every day is consistent with the well-documented clustering of health-promoting and health-compromising behaviors during adolescence [4] and with GSHS-based analyses elsewhere in which physical inactivity accompanied inadequate VF intake [16]. In contrast, the association with soft drink consumption warrants cautious interpretation: it was non-monotonic, with moderate consumption associated with a lower, and very frequent consumption (≥3 times per day) with a borderline higher, prevalence of the outcome. This pattern may reflect a subgroup of adolescents with generally high reported consumption frequency across all food and beverage categories, reporting tendencies inherent to frequency-based self-report items, or residual confounding.

This study has several strengths, including the use of nationally representative samples from five GCC countries, harmonized outcome and covariate definitions across surveys, full incorporation of the complex survey design with sampling weights, and the estimation of prevalence ratios, which are more interpretable than odds ratios for common outcomes. Several limitations should also be acknowledged. The cross-sectional design precludes causal inference, and all measures were self-reported. VF intake was assessed by single-frequency items that do not capture portion sizes or allow quantification in grams. Therefore, the indicator used here is a proxy for, and not a measure of, the WHO target of at least 400 g per day. The GSHS also contains no measure of household income, parental occupation, or parental education, so the single hunger item is the only available proxy for socioeconomic position. The null association observed for food insecurity should not be read as evidence that socioeconomic position is unrelated to adolescent diet in the Gulf. The survey years differ across countries, and the Qatar data (2011) in particular predate the other surveys by four to five years, so cross-country comparisons should be interpreted with caution. More broadly, the surveys were fielded between 2011 and 2016, so the estimates describe that period and should not be read as current prevalence. National nutrition policies, school food environments, food availability, the use of food-delivery applications, the consumption of ultra-processed foods, and dietary behavior following the COVID-19 pandemic may all have changed in the region since the data were collected. Saudi Arabia, the most populous GCC country, could not be included because GSHS data were unavailable, and weight status could not be modeled because anthropometric data were missing for Qatar. Finally, the findings generalize only to school-going adolescents and not to out-of-school youth.

The findings have practical implications for adolescent nutrition policy in the Gulf region. School-based nutrition interventions in Arab League states have demonstrated effectiveness in improving dietary behaviors when they combine curriculum-based education with changes to the school food environment [18]. In addition, WHO Eastern Mediterranean Region reviews document existing national platforms, including school canteen standards and fruit and vegetable schemes, in GCC countries upon which such programs can build [19]. Digital approaches also show promise. A randomized controlled trial of a smartphone application improved VF consumption among Saudi adolescents [20]. Stakeholder-engaged priority setting in the region further emphasizes reshaping the school food environment as an actionable lever [21]. Given the patterns observed here, interventions should engage families, give particular attention to girls, and intensify support across the adolescent years to counteract the age-related decline in VF consumption.

## 5. Conclusions

In this pooled analysis of nationally representative GSHS data from five GCC countries, fewer than one in four school-going adolescents consumed VF five or more times per day, with the lowest prevalence in Kuwait and the highest in Qatar. Boys consistently had a higher prevalence than girls, and consumption declined sharply with age. Low parental support was independently associated with lower consumption, especially among girls. These findings support multicomponent school-based nutrition programs that engage families, target girls and older adolescents, and strengthen existing national school food policies. Continued GSHS surveillance with a new GSHS wave in the region, including Saudi Arabia, is needed to establish current levels and to monitor progress.

## Figures and Tables

**Table 1:** Characteristics of participants by country (unweighted numbers and percentages).

	Bahrain	Kuwait	Oman	Qatar	UAE	Total
Sample (unweighted)	7037 (32.7%)	3534 (16.4%)	3376 (15.7%)	1860 (8.6%)	5721 (26.6%)	21,528 (100.0%)
Age						
12 or younger	1181 (16.8%)	80 (2.3%)	74 (2.2%)	470 (25.5%)	308 (5.4%)	2113 (9.8%)
13–15	4281 (60.9%)	1943 (55.4%)	1589 (47.2%)	1311 (71.1%)	3121 (54.8%)	12,245 (57.1%)
16–18	1573 (22.4%)	1484 (42.3%)	1704 (50.6%)	62 (3.4%)	2271 (39.8%)	7094 (33.1%)
Sex						
Girls	3417 (48.6%)	1866 (53.8%)	1748 (52.6%)	1004 (54.6%)	3008 (52.9%)	11,043 (51.7%)
Boys	3615 (51.4%)	1602 (46.2%)	1574 (47.4%)	835 (45.4%)	2681 (47.1%)	10,307 (48.3%)
Fruit consumption						
None	399 (5.7%)	485 (13.7%)	181 (5.4%)	294 (15.8%)	352 (6.2%)	1711 (7.9%)
≤1 time/day	4149 (59.0%)	2024 (57.3%)	1950 (57.8%)	848 (45.6%)	3489 (61.0%)	12,460 (57.9%)
2–3 times/day	1808 (25.7%)	857 (24.3%)	847 (25.1%)	453 (24.4%)	1394 (24.4%)	5359 (24.9%)
≥4 times/day	681 (9.7%)	168 (4.8%)	398 (11.8%)	265 (14.2%)	486 (8.5%)	1998 (9.3%)
Vegetable consumption						
None	517 (7.3%)	322 (9.1%)	263 (7.8%)	279 (15.0%)	406 (7.1%)	1787 (8.3%)
≤1 time/day	3857 (54.8%)	1961 (55.5%)	2036 (60.3%)	887 (47.7%)	3247 (56.8%)	11,988 (55.7%)
2–3 times/day	1870 (26.6%)	980 (27.7%)	731 (21.7%)	455 (24.5%)	1440 (25.2%)	5476 (25.4%)
≥4 times/day	793 (11.3%)	271 (7.7%)	346 (10.2%)	239 (12.8%)	628 (11.0%)	2277 (10.6%)
Food insecurity						
Never	3186 (45.3%)	1805 (51.3%)	2080 (61.9%)	1288 (70.7%)	2926 (51.3%)	11,285 (52.7%)
Rarely/sometimes	3182 (45.3%)	1451 (41.2%)	1120 (33.3%)	411 (22.5%)	2205 (38.7%)	8369 (39.1%)
Most of the time/always	658 (9.4%)	263 (7.5%)	159 (4.7%)	124 (6.8%)	571 (10.0%)	1775 (8.3%)
Low parental support						
No	6314 (91.4%)	2546 (80.6%)	2730 (85.6%)	1067 (68.6%)	4850 (88.9%)	17,507 (86.4%)
Yes	595 (8.6%)	613 (19.4%)	461 (14.4%)	489 (31.4%)	608 (11.1%)	2766 (13.6%)
Physically active ≥ 60 min/day on all 7 days						
Yes	1454 (20.7%)	497 (15.3%)	374 (11.3%)	175 (10.1%)	869 (15.5%)	3369 (16.1%)
No	5559 (79.3%)	2758 (84.7%)	2944 (88.7%)	1562 (89.9%)	4749 (84.5%)	17,572 (83.9%)
Soft drink consumption						
0 times/day	1744 (24.9%)	623 (17.8%)	676 (20.2%)	257 (14.0%)	1063 (18.8%)	4363 (20.4%)
<1 time/day	3052 (43.5%)	1038 (29.6%)	1232 (36.8%)	464 (25.3%)	2592 (45.8%)	8378 (39.2%)
1–2 times/day	1598 (22.8%)	1290 (36.8%)	1004 (30.0%)	625 (34.0%)	1367 (24.2%)	5884 (27.6%)
≥3 times/day	616 (8.8%)	554 (15.8%)	436 (13.0%)	490 (26.7%)	634 (11.2%)	2730 (12.8%)
Fast food consumption						
0 days	1815 (25.8%)	849 (24.1%)	963 (28.6%)	252 (13.9%)	1320 (23.1%)	5199 (24.3%)
1–3 days	4222 (60.1%)	2147 (61.1%)	1964 (58.3%)	1085 (59.8%)	3688 (64.5%)	13,106 (61.1%)
4–7 days	987 (14.1%)	520 (14.8%)	440 (13.1%)	478 (26.3%)	706 (12.4%)	3131 (14.6%)

Column percentages are based on non-missing values within each country; category totals may differ from the overall sample size because of item-level missing data. UAE = United Arab Emirates.

**Table 2:** Weighted prevalence (%) of vegetables and fruit consumption frequency and of consuming vegetables and fruit on five or more occasions per day among adolescents in GCC countries, overall and by sex.

	Bahrain (2016)	Kuwait (2015)	Oman (2015)	Qatar (2011)	UAE (2016)
**Overall**					
Fruit consumption					
None	6.03 (5.20–6.99)	14.10 (11.85–16.70)	5.38 (4.20–6.86)	16.17 (14.15–18.42)	5.85 (4.86–7.02)
≤1 time/day	61.73 (59.74–63.68)	57.24 (53.90–60.51)	57.81 (55.06–60.52)	45.58 (42.77–48.41)	59.36 (55.14–63.45)
2–3 times/day	21.58 (20.31–22.91)	23.82 (21.01–26.87)	25.01 (23.00–27.14)	24.02 (21.78–26.41)	26.42 (23.06–30.08)
≥4 times/day	10.66 (9.48–11.96)	4.84 (4.07–5.75)	11.80 (10.10–13.73)	14.23 (12.30–16.41)	8.37 (7.10–9.85)
Vegetable consumption					
None	8.20 (7.28–9.22)	9.37 (8.00–10.94)	7.97 (6.80–9.31)	15.37 (13.38–17.58)	6.48 (5.69–7.37)
≤1 time/day	59.62 (57.84–61.36)	55.41 (52.14–58.63)	59.69 (57.03–62.29)	47.45 (44.52–50.41)	55.19 (50.88–59.44)
2–3 times/day	20.87 (20.05–21.71)	27.67 (24.38–31.22)	21.99 (19.88–24.24)	24.23 (22.28–26.30)	27.28 (23.05–31.95)
≥4 times/day	11.32 (10.06–12.71)	7.56 (6.53–8.73)	10.36 (9.14–11.72)	12.95 (10.77–15.50)	11.05 (10.02–12.17)
Consumed vegetables and fruit ≥ 5 times/day	20.99 (19.24–22.85)	15.49 (13.23–18.05)	21.46 (19.20–23.91)	25.21 (22.62–27.99)	22.11 (19.45–25.02)
**Girls**					
Fruit consumption					
None	5.72 (4.63–7.06)	14.28 (10.55–19.04)	5.32 (3.71–7.57)	15.11 (12.82–17.74)	5.32 (3.94–7.14)
≤1 time/day	65.72 (63.49–67.89)	58.18 (53.86–62.39)	62.68 (59.59–65.66)	47.10 (42.88–51.36)	63.38 (58.91–67.64)
2–3 times/day	19.69 (17.79–21.75)	23.60 (19.47–28.30)	22.70 (20.22–25.39)	25.11 (21.68–28.89)	24.85 (20.79–29.42)
≥4 times/day	8.86 (7.24–10.80)	3.94 (3.42–4.53)	9.31 (7.63–11.31)	12.67 (10.16–15.71)	6.45 (5.15–8.04)
Vegetable consumption					
None	7.32 (5.91–9.04)	9.34 (7.31–11.86)	8.07 (6.57–9.87)	14.57 (12.36–17.10)	6.28 (5.18–7.61)
≤1 time/day	62.46 (59.77–65.07)	55.98 (51.96–59.92)	64.69 (62.27–67.04)	49.61 (45.79–53.44)	55.21 (49.18–61.09)
2–3 times/day	20.20 (18.31–22.24)	27.18 (23.67–31.01)	18.04 (16.44–19.76)	24.60 (22.15–27.23)	28.78 (22.81–35.60)
≥4 times/day	10.02 (8.68–11.53)	7.50 (6.04–9.28)	9.21 (7.58–11.14)	11.21 (8.98–13.92)	9.72 (8.41–11.22)
Consumed vegetables and fruit ≥ 5 times/day	18.56 (16.92–20.31)	14.78 (11.93–18.15)	17.77 (15.24–20.62)	22.57 (19.31–26.21)	20.95 (17.65–24.68)
**Boys**					
Fruit consumption					
None	6.34 (5.29–7.58)	13.62 (10.77–17.09)	5.04 (3.87–6.54)	16.96 (13.51–21.08)	6.32 (5.20–7.65)
≤1 time/day	57.83 (55.17–60.45)	56.66 (52.83–60.41)	52.98 (49.64–56.29)	44.29 (40.68–47.96)	55.34 (50.68–59.91)
2–3 times/day	23.42 (21.08–25.94)	24.12 (20.94–27.62)	27.63 (24.86–30.59)	22.91 (19.90–26.24)	27.92 (24.09–32.10)
≥4 times/day	12.41 (11.06–13.90)	5.60 (4.37–7.13)	14.35 (12.06–16.99)	15.84 (12.90–19.30)	10.42 (8.96–12.08)
Vegetable consumption					
None	9.06 (8.00–10.23)	9.29 (7.76–11.08)	7.84 (6.08–10.06)	16.15 (12.84–20.12)	6.66 (5.48–8.07)
≤1 time/day	56.82 (54.87–58.75)	54.90 (51.23–58.52)	54.47 (51.27–57.63)	45.17 (41.05–49.37)	55.15 (51.02–59.22)
2–3 times/day	21.54 (19.47–23.76)	28.26 (24.47–32.40)	26.12 (23.49–28.94)	24.07 (20.90–27.55)	25.80 (21.95–30.06)
≥4 times/day	12.58 (11.06–14.29)	7.55 (6.23–9.11)	11.56 (9.98–13.37)	14.61 (11.11–18.97)	12.39 (11.21–13.67)
Consumed vegetables and fruit ≥ 5 times/day	23.36 (21.25–25.62)	15.87 (13.02–19.21)	25.21 (22.50–28.14)	28.18 (24.38–32.32)	23.25 (20.52–26.23)

Values are weighted % (95% CI). Vegetable and Fruit consumption categories derived from GSHS items: None = did not eat during the past 30 days; ≤1 time/day = less than once to once per day; 2–3 times/day = two to three times per day; ≥4 times/day = four or more times per day. Consumed vegetables and fruit ≥ 5 times/day = a combined vegetables and fruit consumption frequency of five or more eating occasions per day. The survey year is shown beneath each country name. All estimates are survey-weighted using the GSHS complex sample design. GCC = Gulf Cooperation Council; CI = confidence interval; UAE = United Arab Emirates.

**Table 3:** Adjusted associations between covariates and consuming vegetables and fruit on five or more occasions per day in the overall sample and stratified by sex.

	Overall aPR (95% CI) *	Girls aPR (95% CI) *	Boys aPR (95% CI) *
Age			
12 years or younger	Ref.	Ref.	Ref.
13–15 years	0.83 (0.72–0.97)	0.84 (0.66–1.08)	0.83 (0.70–0.98)
16–18 years	0.66 (0.56–0.79)	0.72 (0.55–0.94)	0.62 (0.50–0.76)
Sex			
Girls	Ref.	—	—
Boys	1.18 (1.08–1.29)	—	—
Food insecurity			
Never	Ref.	Ref.	Ref.
Rarely/sometimes	0.94 (0.87–1.02)	0.98 (0.89–1.08)	0.91 (0.82–1.01)
Most of the time/always	1.07 (0.94–1.23)	0.96 (0.78–1.19)	1.15 (0.98–1.35)
Physically active (all 7 days)			
Yes	Ref.	Ref.	Ref.
No	0.68 (0.62–0.74)	0.64 (0.55–0.74)	0.71 (0.63–0.79)
Fast food consumption			
0 days	Ref.	Ref.	Ref.
1–3 days	0.93 (0.85–1.03)	0.93 (0.83–1.04)	0.95 (0.83–1.08)
4–7 days	0.96 (0.84–1.10)	0.82 (0.69–0.97)	1.10 (0.91–1.33)
Soft drink consumption			
0 times/day	Ref.	Ref.	Ref.
<1 time/day	0.64 (0.59–0.70)	0.67 (0.59–0.76)	0.62 (0.54–0.71)
1–2 times/day	0.70 (0.64–0.78)	0.78 (0.67–0.91)	0.65 (0.56–0.75)
≥3 times/day	1.13 (1.00–1.27)	1.18 (0.99–1.41)	1.08 (0.90–1.30)
Low parental support			
No	Ref.	Ref.	Ref.
Yes	0.85 (0.76–0.95)	0.73 (0.61–0.86)	0.94 (0.81–1.08)

* Adjusted prevalence ratios (aPRs) from survey-weighted Poisson regression models with a log link. All models were adjusted for all variables in the table in addition to country. aPR = adjusted prevalence ratio; CI = confidence interval; Ref. = reference category. The sex by low parental support interaction term fitted in the pooled model was statistically significant (*p* = 0.019).

## Data Availability

The GSHS datasets analyzed in this study are publicly available from the World Health Organization NCD Microdata Repository at https://extranet.who.int/ncdsmicrodata/index.php/home. accessed on 3 July 2026.

## Supplementary Materials

The following supporting information can be downloaded at https://www.mdpi.com/article/10.3390/nu18172783/s1. Table S1: Global School-based Student Health Survey (GSHS) items, response options, and analytic coding of the outcome and covariates; Table S2: Crude and age-standardized weighted prevalence (%) of consuming vegetables and fruit ≥ 5 times per day, by country; Table S3: Adjusted prevalence ratios for consuming vegetables and fruit ≥ 5 times per day under three specifications of physical activity.

## References

[1] World Health Organization (2003). Diet, Nutrition, and the Prevention of Chronic Diseases: Report of a Joint WHO/FAO Expert Consultation.

[2] World Health Organization (2023). Carbohydrate Intake for Adults and Children: WHO Guideline.

[3] Tian Y., Su L., Wang J., Duan X., Jiang X. (2018). Fruit and vegetable consumption and risk of the metabolic syndrome: A meta-analysis. Public Health Nutr..

[4] Efa Y.T., Roder D., Shi Z., Li M. (2025). Clustering Patterns of Unhealthy Lifestyle Behaviours Among Adolescents: A Multilevel Analysis of a Nationally Representative School-Based Survey from 73 Countries. Nutrients.

[5] Al Makadma A.S. (2017). Adolescent health and health care in the Arab Gulf countries: Today’s needs and tomorrow’s challenges. Int. J. Pediatr. Adolesc. Med..

[6] Zeidan W., Taweel H., Shalash A., Husseini A. (2023). Consumption of fruits and vegetables among adolescents in Arab Countries: A systematic review. Int. J. Behav. Nutr. Phys. Act..

[7] Alasqah I., Mahmud I., East L., Usher K. (2021). Patterns of physical activity and dietary habits among adolescents in Saudi Arabia: A systematic review. Int. J. Health Sci..

[8] Makansi N., Allison P., Awad M., Bedos C. (2018). Fruit and vegetable intake among Emirati adolescents: A mixed methods study. East. Mediterr. Health J..

[9] Pengpid S., Peltzer K. (2020). Trends in the prevalence of twenty health indicators among adolescents in United Arab Emirates: Cross-sectional national school surveys from 2005, 2010 and 2016. BMC Pediatr..

[10] Achak D., Azizi A., El-Ammari A., Marfak I.Y., Saad E., Nejjari C., Hilali A., Peltzer K., Marfak A. (2024). The health behaviors differences among male and female school-age adolescents in the Middle East and North Africa region countries: A meta-analysis of the Global School-based Student Health Survey data. Front. Public Health.

[11] World Health Organization Global School-Based Student Health Survey (GSHS). https://www.who.int/teams/noncommunicable-diseases/surveillance/systems-tools/global-school-based-student-health-survey.

[12] Al-Zalabani A.H. (2024). Prevalence and Predictors of Soft Drink Consumption among Adolescents in the Gulf Countries: Findings from National Surveys. Nutrients.

[13] Al-Zalabani A.H. (2025). The association between cigarette smoking and sleep deprivation among adolescents in Gulf Cooperation Council countries: Analysis of national surveys. Neurosciences.

[14] Hall S., Moskovitz C., Pemberton M. Understanding Text Recycling: A Guide for Researchers. V1.1 April, 2021. https://textrecycling.org/files/2021/06/Understanding-Text-Recycling_A-Guide-for-Researchers-V.1.pdf.

[15] Hoteit M., Hallit S., Al Rawas H., Amasha J., Kobeissi F., Fayyad R., Sacre Y., Tzenios N. (2024). Adolescent Health in Lebanon: Exploring Alcohol Use, Dietary Patterns, Mental Health, Physical Activity, and Smoking Using the Global School-Based Student Health Survey Approach. Nutrients.

[16] Pengpid S., Peltzer K. (2020). Prevalence and correlates of fruit and vegetable consumption among adolescents in Laos. Int. J. Adolesc. Med. Health.

[17] Smith L., Sánchez G.F.L., Tully M.A., Rahmati M., Oh H., Kostev K., Butler L.T., Barnett Y., Keyes H., Shin J.I. (2024). Temporal trends of carbonated soft-drink consumption among adolescents aged 12–15 years from eighteen countries in Africa, Asia and the Americas. Br. J. Nutr..

[18] Abu Shihab K.H.N., Dodge E., Benajiba N., Chavarria E.A., Aboul-Enein B.H., Faris M.A.-I.E. (2023). Effectiveness of school-based nutrition interventions promoted in the League of Arab States: A systematic review. Health Promot. Int..

[19] Al-Jawaldeh A., Matbouli D., Diab S., Taktouk M., Hojeij L., Naalbandian S., Nasreddine L. (2023). School-Based Nutrition Programs in the Eastern Mediterranean Region: A Systematic Review. Int. J. Environ. Res. Public Health.

[20] Shatwan I.M., Alhefani R.S., Bukhari M.F., Hanbazazah D.A., Srour J.K., Surendran S., Aljefree N.M., Almoraie N.M. (2023). Effects of a Smartphone App on Fruit and Vegetable Consumption Among Saudi Adolescents: Randomized Controlled Trial. JMIR Pediatr. Parent..

[21] Almughamisi M., O’Keeffe M., Harding S. (2022). Adolescent Obesity Prevention in Saudi Arabia: Co-identifying Actionable Priorities for Interventions. Front. Public Health.

