# Supplementary material for: Vegetable and Fruit Consumption Among Adolescents in the Gulf Cooperation Council Countries: A Pooled Analysis of National School Surveys
Source: Nutrients. 2026 Aug 26;18(17):2783. doi: 10.3390/nu18172783 (PMC13566987; doi:10.3390/nu18172783)
Supplement: Supplementary file 1 [file nutrients-18-02783-s001.zip › nutrients-4449060-supplementary.pdf]

Supplementary Materials

Table S1. Global School-based Student Health Survey (GSHS) items, response options, and analytic coding of the outcome and covariates.

Variable	GSHS item	Response options	Analytic coding
**Age**	How old are you?	11 years old or younger, 12, 13, 14, 15, 16, 17, and 18 years old or older	Three groups: ≤12, 13–15, 16–18 years
**Sex**	What is your sex?	Male; Female	Binary; used as a covariate and as a stratifying variable
**Fruit consumption**	During the past 30 days, how many times per day did you usually eat fruit, such as dates, bananas, apples, oranges, mangos, berries, or any other fruits?	I did not eat fruit during the past 30 days; less than 1 time/day; 1; 2; 3; 4; 5 or more times/day	Descriptive: none; ≤1 time/day; 2–3 times/day; ≥4 times/day. Also entered into the combined VF score
**Vegetable consumption**	During the past 30 days, how many times per day did you usually eat vegetables, such as lettuce, cucumbers, tomatoes, carrots, or any other vegetables?	I did not eat vegetables during the past 30 days; less than 1 time/day; 1; 2; 3; 4; 5 or more times/day	Descriptive: none; ≤1 time/day; 2–3 times/day; ≥4 times/day. Also entered into the combined VF score
**Primary outcome:** Vegetables and fruit, five or more occasions per day	Derived from the two items above	—	Vegetables and Fruit frequencies summed; binary indicator of ≥5 occasions/day (yes/no)
**Food insecurity**	During the past 30 days, how often did you go hungry because there was not enough food in your home?	Never; rarely; sometimes; most of the time; always	Three groups: never; rarely/sometimes; most of the time/always
**Physical activity**	During the past 7 days, on how many days were you physically active for a total of at least 60 minutes per day? ADD UP ALL THE TIME YOU SPENT IN ANY KIND OF PHYSICAL ACTIVITY EACH DAY. *Item preamble: "Physical activity is any activity that increases your heart rate and makes you get out of breath some of the time. Physical activity can be done in sports, playing with friends, or walking to school. Some examples of physical activity are running, fast walking, biking, dancing, football, swimming, and aerobics."*	0 to 7 days	Primary models: active ≥60 min/day on all 7 days (yes/no). Sensitivity: 5 graded groups and a linear days/week term (Tables S3, S3b)
**Soft drink consumption**	During the past 30 days, how many times per day did you usually drink carbonated soft drinks, such as Pepsi, Coca Cola, 7 Up, Sprite, Mountain Dew, Fanta, or any other carbonated soft drink? (Do not include diet soft drinks.)	I did not drink carbonated soft drinks during the past 30 days; less than 1 time/day; 1; 2; 3; 4; 5 or more times/day	Four groups: 0; <1; 1–2; ≥3 times/day
**Fast food consumption**	During the past 7 days, on how many days did you eat food from a fast food restaurant?	0 to 7 days	Three groups: 0; 1–3; 4–7 days
**Low parental support**	During the past 30 days, how often did your parents or guardians (a) check to see if your homework was done, (b)	Never; rarely; sometimes; most of the time; always (each item)	Low support = responded "rarely" or "never" to all

Variable	GSHS item	Response options	Analytic coding
	understand your problems and worries, (c) really know what you were doing with your free time?		three items; otherwise adequate support

VF = Vegetables and Fruit. All items are self-reported and refer to the recall period stated in the item.

Table S2. Crude and age-standardized weighted prevalence (%) of consuming vegetables and fruit ≥5 times per day, by country.

Country	Survey year	Crude % (95% CI)	Age-standardized % (95% CI)	Difference (pp)[a]
Bahrain	2016	21.0 (19.2–22.9)	19.9 (18.4–21.5)	−1.1
Kuwait	2015	15.5 (13.2–18.1)	15.7 (13.5–17.9)	+0.2
Oman	2015	21.5 (19.2–23.9)	21.7 (19.3–24.2)	+0.2
Qatar	2011	25.2 (22.6–28.0)	26.6 (22.1–31.1)	+1.4
UAE	2016	22.1 (19.5–25.0)	22.1 (19.5–24.6)	0.0

Estimates are survey-weighted and account for the GSHS complex sample design (weights, primary sampling units, and strata). Age standardization uses the direct method with the pooled weighted age distribution of the five countries as the standard population.

[a] Age-standardized minus crude prevalence, in percentage points. CI = confidence interval; pp = percentage points; UAE = United Arab Emirates.

Table S3. Adjusted prevalence ratios for consuming vegetables and fruit ≥5 times per day under three specifications of physical activity.

Covariate	Model 1<br>PA dichotomous<br>(primary)	Model 2<br>PA graded, 5 groups	Model 3<br>PA linear, days/week
**Country**			
Bahrain	1.00 (Ref.)	1.00 (Ref.)	1.00 (Ref.)
Kuwait	0.80 (0.67–0.95)	0.79 (0.66–0.93)	0.79 (0.67–0.94)
Oman	1.12 (0.98–1.28)	1.16 (1.01–1.32)	1.16 (1.02–1.33)
Qatar	1.11 (0.98–1.26)	1.16 (1.02–1.32)	1.16 (1.03–1.32)
United Arab Emirates	1.13 (1.00–1.27)	1.10 (0.98–1.24)	1.12 (0.99–1.25)
**Age group**			
12 years or younger	1.00 (Ref.)	1.00 (Ref.)	1.00 (Ref.)
13–15 years	0.83 (0.72–0.97)	0.85 (0.73–0.99)	0.85 (0.73–0.99)
16–18 years	0.66 (0.56–0.79)	0.69 (0.58–0.82)	0.68 (0.57–0.81)
**Sex**			
Girls	1.00 (Ref.)	1.00 (Ref.)	1.00 (Ref.)
Boys	1.18 (1.08–1.29)	1.15 (1.05–1.25)	1.14 (1.05–1.25)
**Food insecurity**			
Never	1.00 (Ref.)	1.00 (Ref.)	1.00 (Ref.)
Rarely/sometimes	0.94 (0.87–1.02)	0.95 (0.88–1.02)	0.95 (0.88–1.02)
Most of the time/always	1.07 (0.94–1.23)	1.09 (0.95–1.25)	1.09 (0.95–1.25)
**Physical activity**			
Active ≥60 min/day on all 7 days	1.00 (Ref.)	1.00 (Ref.)	—
Not active on all 7 days	0.68 (0.62–0.74)	—	—
5–6 days	—	0.97 (0.86–1.10)	—
3–4 days	—	0.87 (0.78–0.97)	—
1–2 days	—	0.58 (0.52–0.65)	—
0 days	—	0.53 (0.47–0.61)	—
Per 1 additional active day/week	—	—	1.10 (1.09–1.12)
**Fast food consumption**			
0 days	1.00 (Ref.)	1.00 (Ref.)	1.00 (Ref.)
1–3 days	0.93 (0.85–1.03)	0.93 (0.85–1.02)	0.93 (0.85–1.02)
4–7 days	0.96 (0.84–1.10)	0.97 (0.85–1.10)	0.97 (0.85–1.10)
**Soft drink consumption**			
0 times/day	1.00 (Ref.)	1.00 (Ref.)	1.00 (Ref.)
<1 time/day	0.64 (0.59–0.70)	0.65 (0.60–0.71)	0.65 (0.60–0.71)
1–2 times/day	0.70 (0.64–0.78)	0.72 (0.65–0.80)	0.72 (0.65–0.80)
≥3 times/day	1.13 (1.00–1.27)	1.16 (1.03–1.30)	1.16 (1.02–1.30)
**Low parental support**			
No	1.00 (Ref.)	1.00 (Ref.)	1.00 (Ref.)
Yes	0.85 (0.76–0.95)	0.89 (0.79–0.99)	0.89 (0.79–0.99)

Values are adjusted prevalence ratios (95% confidence intervals) from survey-weighted Poisson regression models with a log link, each mutually adjusted for all variables shown. Model 1 is the specification reported in the main analysis (Table 3), in which physical activity is

dichotomized as active for at least 60 minutes per day on all 7 days versus not. Model 2 replaces this with five graded groups of active days per week. Model 3 replaces it with a linear term for the number of active days per week.

Estimates for all non-physical-activity covariates are materially unchanged across the three specifications, indicating that the conclusions of the main analysis are not sensitive to how physical activity is operationalized. A dose–response gradient is evident in Models 2 and 3: each additional active day per week is associated with approximately 10% higher prevalence.

— = not applicable to this specification. aPR = adjusted prevalence ratio; PA = physical activity; Ref. = reference category.
